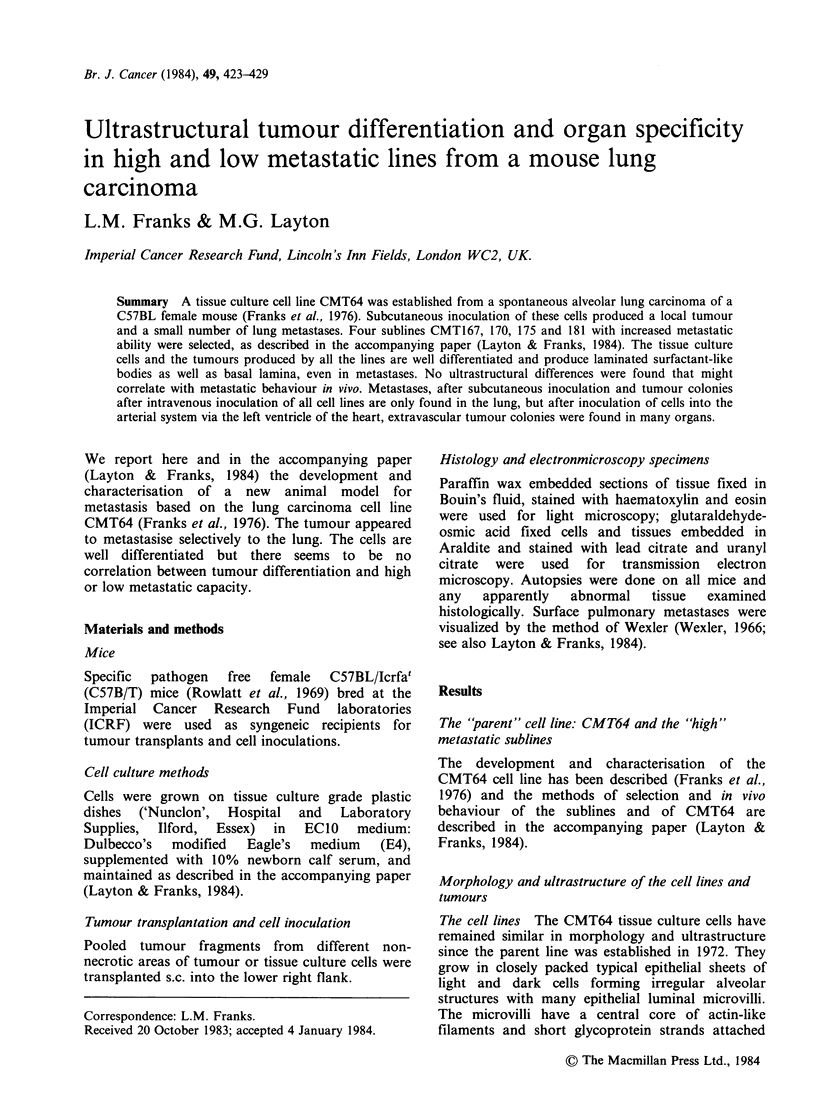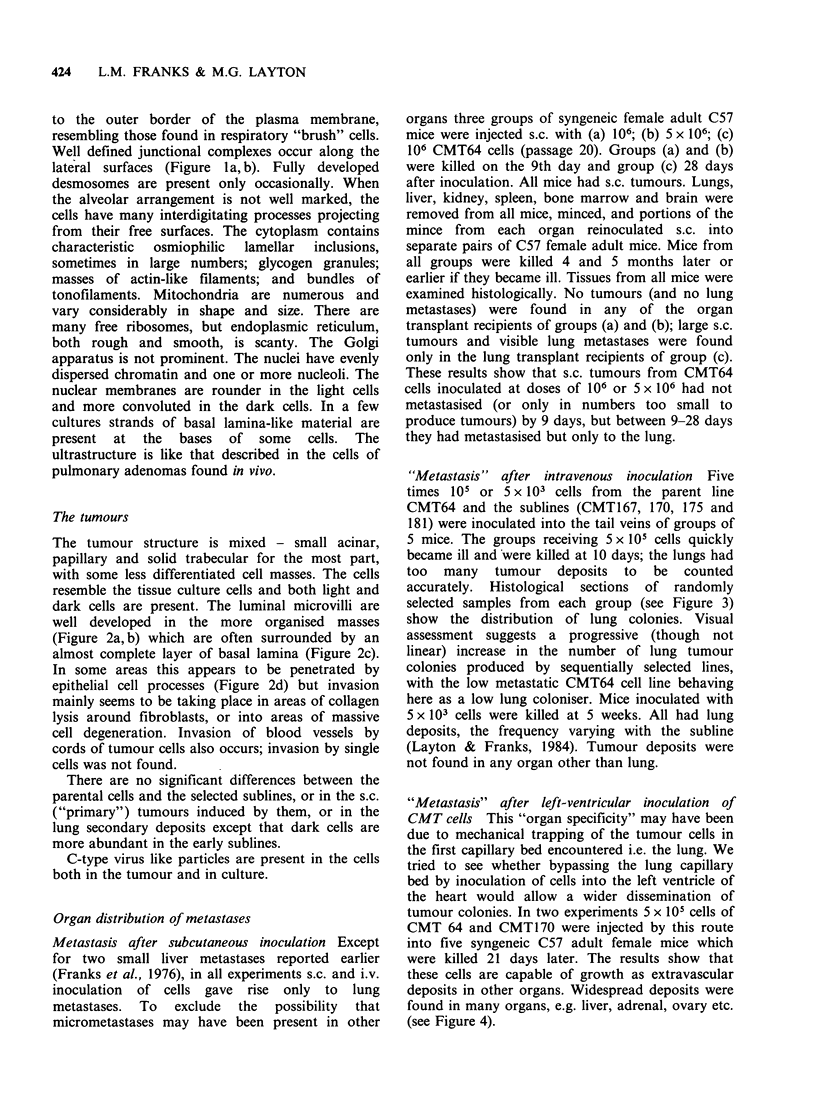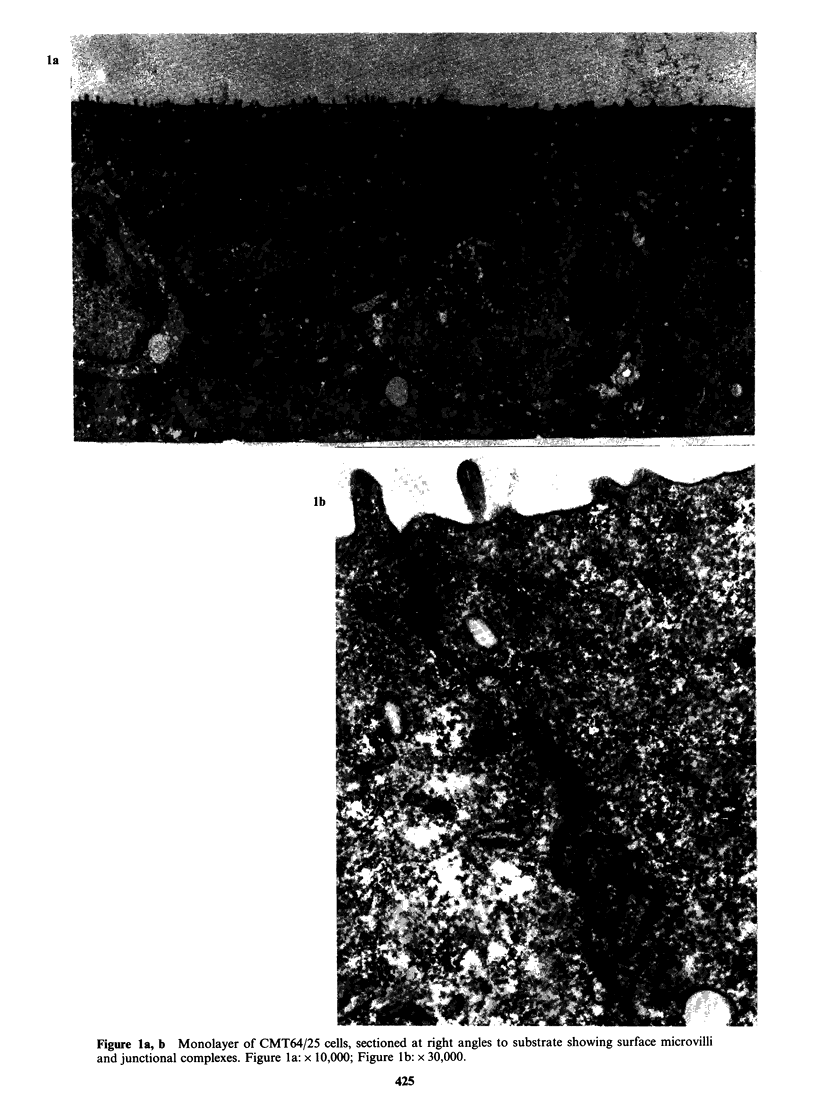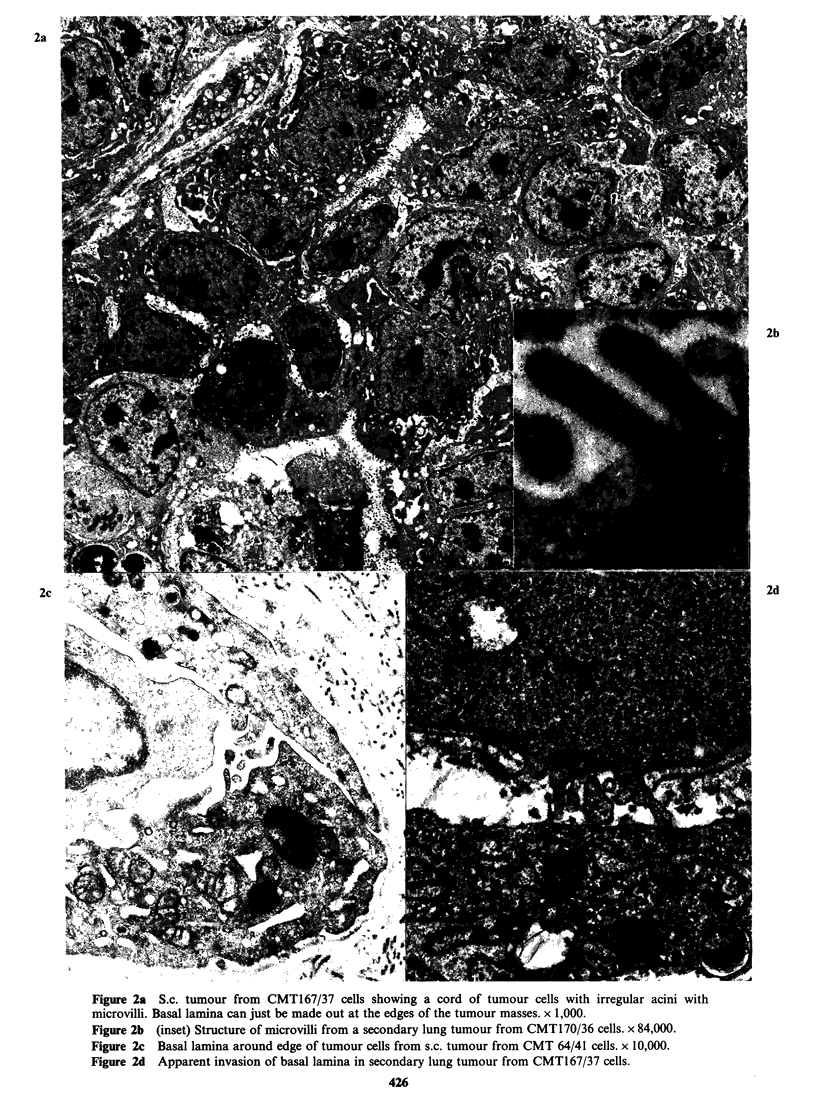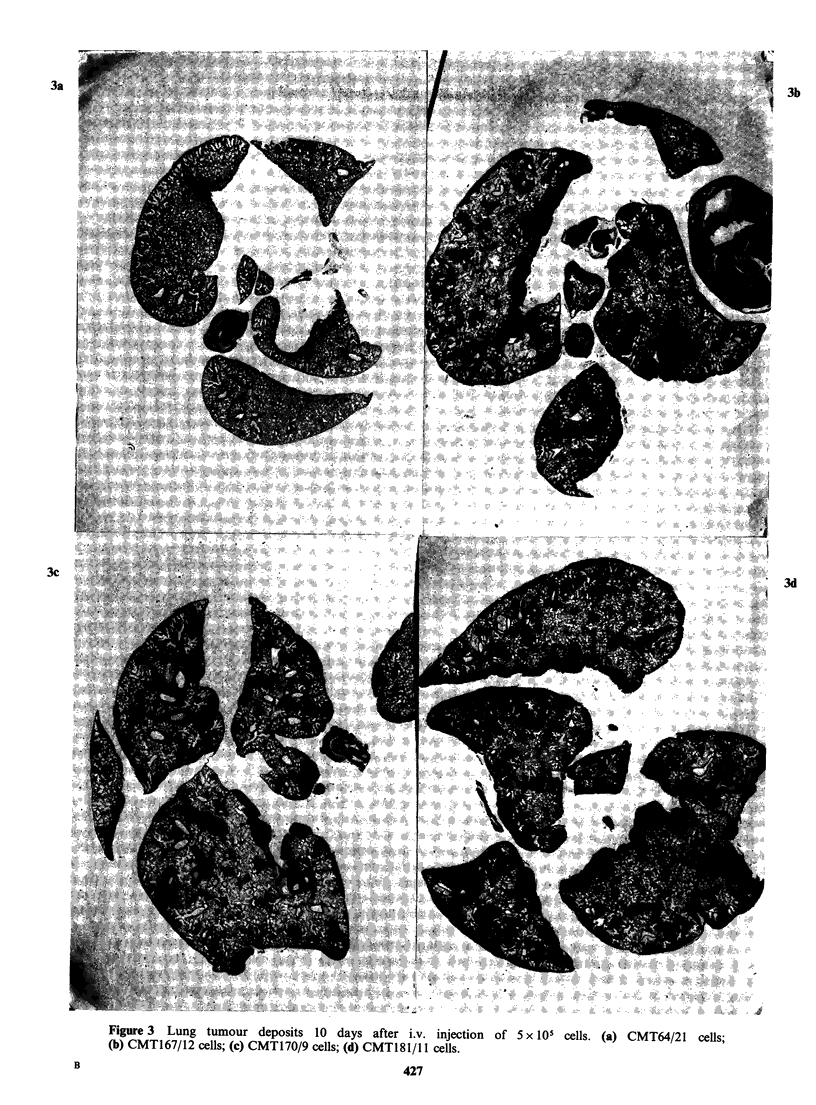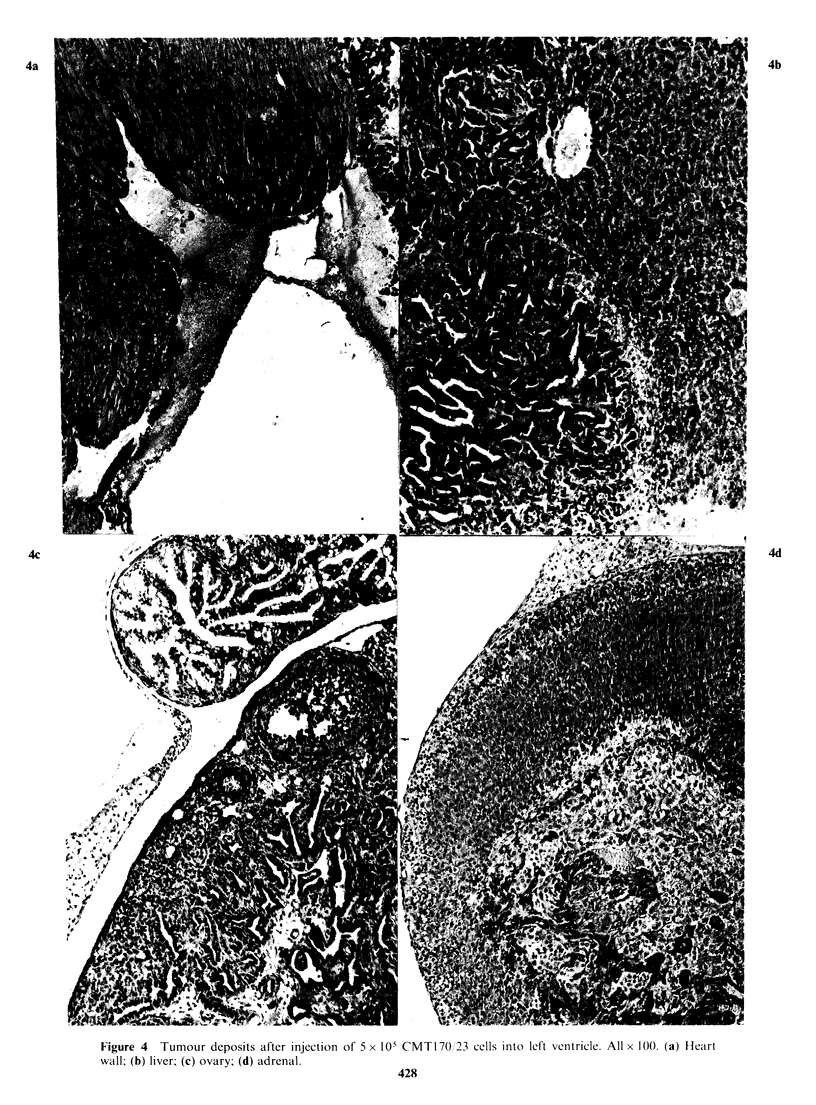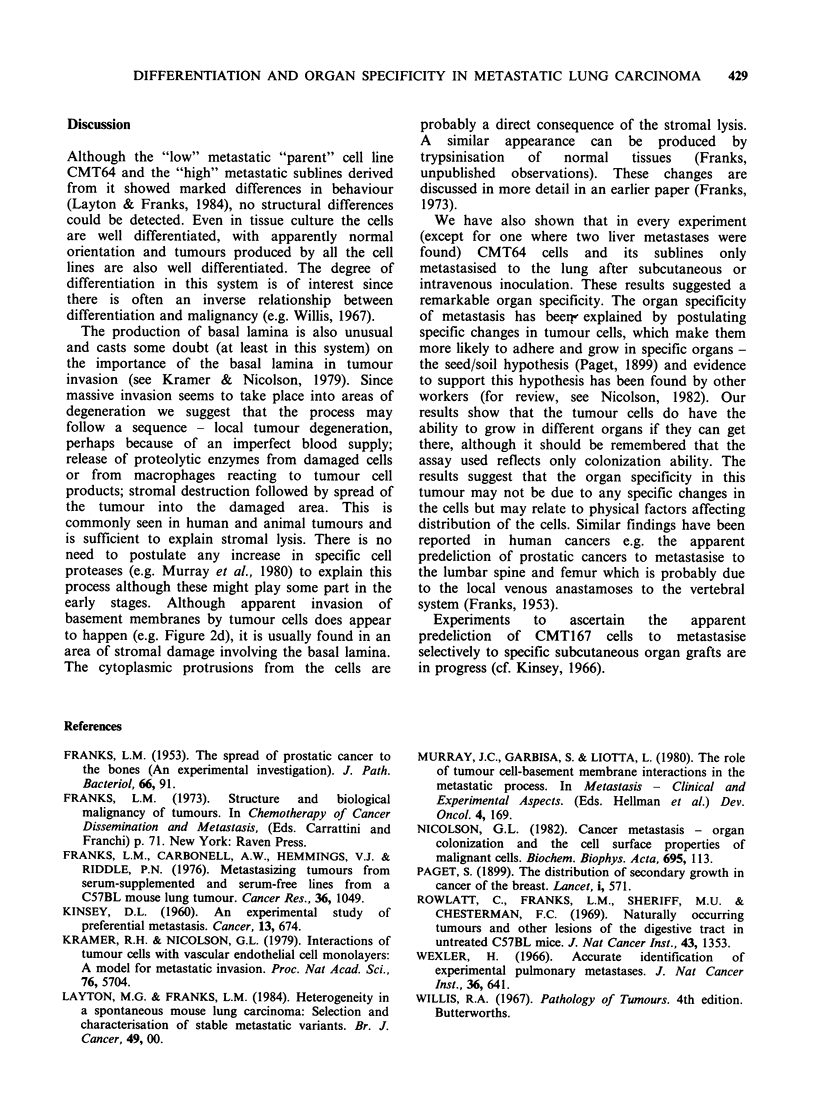# Ultrastructural tumour differentiation and organ specificity in high and low metastatic lines from a mouse lung carcinoma.

**DOI:** 10.1038/bjc.1984.68

**Published:** 1984-04

**Authors:** L. M. Franks, M. G. Layton

## Abstract

**Images:**


*Br. J. Cancer* (1984), **49**, 423–429

### Ultrastructural tumour differentiation and organ specificity in high and low metastatic lines from a mouse lung carcinoma

L.M. Franks & M.G. Layton

*Imperial Cancer Research Fund, Lincoln's Inn Fields, London WC2, UK.*

**Summary** A tissue culture cell line CMT64 was established from a spontaneous alveolar lung carcinoma of a C57BL female mouse (Franks *et al.*, 1976). Subcutaneous inoculation of these cells produced a local tumour and a small number of lung metastases. Four sublines CMT167, 170, 175 and 181 with increased metastatic ability were selected, as described in the accompanying paper (Layton & Franks, 1984). The tissue culture cells and the tumours produced by all the lines are well differentiated and produce laminated surfactant-like bodies as well as basal lamina, even in metastases. No ultrastructural differences were found that might correlate with metastatic behaviour *in vivo*. Metastases, after subcutaneous inoculation and tumour colonies after intravenous inoculation of all cell lines are only found in the lung, but after inoculation of cells into the arterial system via the left ventricle of the heart, extravascular tumour colonies were found in many organs.

We report here and in the accompanying paper (Layton & Franks, 1984) the development and characterisation of a new animal model for metastasis based on the lung carcinoma cell line CMT64 (Franks *et al.*, 1976). The tumour appeared to metastasise selectively to the lung. The cells are well differentiated but there seems to be no correlation between tumour differentiation and high or low metastatic capacity.

### Materials and methods

### *Mice*

Specific pathogen free female C57BL/Icrfa[t] (C57B/T) mice (Rowlatt *et al.*, 1969) bred at the Imperial Cancer Research Fund laboratories (ICRF) were used as syngeneic recipients for tumour transplants and cell inoculations.

### *Cell culture methods*

Cells were grown on tissue culture grade plastic dishes ('Nunclon', Hospital and Laboratory Supplies, Ilford, Essex) in EC10 medium: Dulbecco's modified Eagle's medium (E4), supplemented with 10% newborn calf serum, and maintained as described in the accompanying paper (Layton & Franks, 1984).

### *Tumour transplantation and cell inoculation*

Pooled tumour fragments from different non-necrotic areas of tumour or tissue culture cells were transplanted s.c. into the lower right flank.

Correspondence: L.M. Franks.
Received 20 October 1983; accepted 4 January 1984.

### *Histology and electronmicroscopy specimens*

Paraffin wax embedded sections of tissue fixed in Bouin's fluid, stained with haematoxylin and eosin were used for light microscopy; glutaraldehyde-osmic acid fixed cells and tissues embedded in Araldite and stained with lead citrate and uranyl citrate were used for transmission electron microscopy. Autopsies were done on all mice and any apparently abnormal tissue examined histologically. Surface pulmonary metastases were visualized by the method of Wexler (Wexler, 1966; see also Layton & Franks, 1984).

### Results

### *The "parent" cell line: CMT64 and the "high" metastatic sublines*

The development and characterisation of the CMT64 cell line has been described (Franks *et al.*, 1976) and the methods of selection and *in vivo* behaviour of the sublines and of CMT64 are described in the accompanying paper (Layton & Franks, 1984).

### *Morphology and ultrastructure of the cell lines and tumours*

*The cell lines* The CMT64 tissue culture cells have remained similar in morphology and ultrastructure since the parent line was established in 1972. They grow in closely packed typical epithelial sheets of light and dark cells forming irregular alveolar structures with many epithelial luminal microvilli. The microvilli have a central core of actin-like filaments and short glycoprotein strands attached

© The Macmillan Press Ltd., 1984

to the outer border of the plasma membrane, resembling those found in respiratory "brush" cells. Well defined junctional complexes occur along the lateral surfaces (Figure 1a, b). Fully developed desmosomes are present only occasionally. When the alveolar arrangement is not well marked, the cells have many interdigitating processes projecting from their free surfaces. The cytoplasm contains characteristic osmiophilic lamellar inclusions, sometimes in large numbers; glycogen granules; masses of actin-like filaments; and bundles of tonofilaments. Mitochondria are numerous and vary considerably in shape and size. There are many free ribosomes, but endoplasmic reticulum, both rough and smooth, is scanty. The Golgi apparatus is not prominent. The nuclei have evenly dispersed chromatin and one or more nucleoli. The nuclear membranes are rounder in the light cells and more convoluted in the dark cells. In a few cultures strands of basal lamina-like material are present at the bases of some cells. The ultrastructure is like that described in the cells of pulmonary adenomas found *in vivo*.

*The tumours*

The tumour structure is mixed – small acinar, papillary and solid trabecular for the most part, with some less differentiated cell masses. The cells resemble the tissue culture cells and both light and dark cells are present. The luminal microvilli are well developed in the more organised masses (Figure 2a, b) which are often surrounded by an almost complete layer of basal lamina (Figure 2c). In some areas this appears to be penetrated by epithelial cell processes (Figure 2d) but invasion mainly seems to be taking place in areas of collagen lysis around fibroblasts, or into areas of massive cell degeneration. Invasion of blood vessels by cords of tumour cells also occurs; invasion by single cells was not found.

There are no significant differences between the parental cells and the selected sublines, or in the s.c. ("primary") tumours induced by them, or in the lung secondary deposits except that dark cells are more abundant in the early sublines.

C-type virus like particles are present in the cells both in the tumour and in culture.

*Organ distribution of metastases*

*Metastasis after subcutaneous inoculation* Except for two small liver metastases reported earlier (Franks *et al.*, 1976), in all experiments s.c. and i.v. inoculation of cells gave rise only to lung metastases. To exclude the possibility that micrometastases may have been present in other organs three groups of syngeneic female adult C57 mice were injected s.c. with (a) $10^6$; (b) $5 \times 10^6$; (c) $10^6$ CMT64 cells (passage 20). Groups (a) and (b) were killed on the 9th day and group (c) 28 days after inoculation. All mice had s.c. tumours. Lungs, liver, kidney, spleen, bone marrow and brain were removed from all mice, minced, and portions of the mince from each organ reinoculated s.c. into separate pairs of C57 female adult mice. Mice from all groups were killed 4 and 5 months later or earlier if they became ill. Tissues from all mice were examined histologically. No tumours (and no lung metastases) were found in any of the organ transplant recipients of groups (a) and (b); large s.c. tumours and visible lung metastases were found only in the lung transplant recipients of group (c). These results show that s.c. tumours from CMT64 cells inoculated at doses of $10^6$ or $5 \times 10^6$ had not metastasised (or only in numbers too small to produce tumours) by 9 days, but between 9–28 days they had metastasised but only to the lung.

*"Metastasis" after intravenous inoculation* Five times $10^5$ or $5 \times 10^3$ cells from the parent line CMT64 and the sublines (CMT167, 170, 175 and 181) were inoculated into the tail veins of groups of 5 mice. The groups receiving $5 \times 10^5$ cells quickly became ill and were killed at 10 days; the lungs had too many tumour deposits to be counted accurately. Histological sections of randomly selected samples from each group (see Figure 3) show the distribution of lung colonies. Visual assessment suggests a progressive (though not linear) increase in the number of lung tumour colonies produced by sequentially selected lines, with the low metastatic CMT64 cell line behaving here as a low lung coloniser. Mice inoculated with $5 \times 10^3$ cells were killed at 5 weeks. All had lung deposits, the frequency varying with the subline (Layton & Franks, 1984). Tumour deposits were not found in any organ other than lung.

*"Metastasis" after left-ventricular inoculation of CMT cells* This "organ specificity" may have been due to mechanical trapping of the tumour cells in the first capillary bed encountered i.e. the lung. We tried to see whether bypassing the lung capillary bed by inoculation of cells into the left ventricle of the heart would allow a wider dissemination of tumour colonies. In two experiments $5 \times 10^5$ cells of CMT 64 and CMT170 were injected by this route into five syngeneic C57 adult female mice which were killed 21 days later. The results show that these cells are capable of growth as extravascular deposits in other organs. Widespread deposits were found in many organs, e.g. liver, adrenal, ovary etc. (see Figure 4).

1a

1b

**Figure 1a, b** Monolayer of CMT64/25 cells, sectioned at right angles to substrate showing surface microvilli and junctional complexes. Figure 1a: × 10,000; Figure 1b: × 30,000.

**Figure 2a** S.c. tumour from CMT167/37 cells showing a cord of tumour cells with irregular acini with microvilli. Basal lamina can just be made out at the edges of the tumour masses. × 1,000.

**Figure 2b** (inset) Structure of microvilli from a secondary lung tumour from CMT170/36 cells. × 84,000.

**Figure 2c** Basal lamina around edge of tumour cells from s.c. tumour from CMT 64/41 cells. × 10,000.

**Figure 2d** Apparent invasion of basal lamina in secondary lung tumour from CMT167/37 cells.

3a 3b 3c 3d

**Figure 3** Lung tumour deposits 10 days after i.v. injection of $5 \times 10^5$ cells. **(a)** CMT64/21 cells; **(b)** CMT167/12 cells; **(c)** CMT170/9 cells; **(d)** CMT181/11 cells.

**Figure 4** Tumour deposits after injection of $5 \times 10^5$ CMT170/23 cells into left ventricle. All $\times$ 100. (**a**) Heart wall; (**b**) liver; (**c**) ovary; (**d**) adrenal.

### Discussion

Although the "low" metastatic "parent" cell line CMT64 and the "high" metastatic sublines derived from it showed marked differences in behaviour (Layton & Franks, 1984), no structural differences could be detected. Even in tissue culture the cells are well differentiated, with apparently normal orientation and tumours produced by all the cell lines are also well differentiated. The degree of differentiation in this system is of interest since there is often an inverse relationship between differentiation and malignancy (e.g. Willis, 1967).

The production of basal lamina is also unusual and casts some doubt (at least in this system) on the importance of the basal lamina in tumour invasion (see Kramer & Nicolson, 1979). Since massive invasion seems to take place into areas of degeneration we suggest that the process may follow a sequence – local tumour degeneration, perhaps because of an imperfect blood supply; release of proteolytic enzymes from damaged cells or from macrophages reacting to tumour cell products; stromal destruction followed by spread of the tumour into the damaged area. This is commonly seen in human and animal tumours and is sufficient to explain stromal lysis. There is no need to postulate any increase in specific cell proteases (e.g. Murray *et al.*, 1980) to explain this process although these might play some part in the early stages. Although apparent invasion of basement membranes by tumour cells does appear to happen (e.g. Figure 2d), it is usually found in an area of stromal damage involving the basal lamina. The cytoplasmic protrusions from the cells are probably a direct consequence of the stromal lysis. A similar appearance can be produced by trypsinisation of normal tissues (Franks, unpublished observations). These changes are discussed in more detail in an earlier paper (Franks, 1973).

We have also shown that in every experiment (except for one where two liver metastases were found) CMT64 cells and its sublines only metastasised to the lung after subcutaneous or intravenous inoculation. These results suggested a remarkable organ specificity. The organ specificity of metastasis has been explained by postulating specific changes in tumour cells, which make them more likely to adhere and grow in specific organs – the seed/soil hypothesis (Paget, 1899) and evidence to support this hypothesis has been found by other workers (for review, see Nicolson, 1982). Our results show that the tumour cells do have the ability to grow in different organs if they can get there, although it should be remembered that the assay used reflects only colonization ability. The results suggest that the organ specificity in this tumour may not be due to any specific changes in the cells but may relate to physical factors affecting distribution of the cells. Similar findings have been reported in human cancers e.g. the apparent predeliction of prostatic cancers to metastasise to the lumbar spine and femur which is probably due to the local venous anastamoses to the vertebral system (Franks, 1953).

Experiments to ascertain the apparent predeliction of CMT167 cells to metastasise selectively to specific subcutaneous organ grafts are in progress (cf. Kinsey, 1966).

### References

FRANKS, L.M. (1953). The spread of prostatic cancer to the bones (An experimental investigation). *J. Path. Bacteriol*, **66**, 91.

FRANKS, L.M. (1973). Structure and biological malignancy of tumours. In *Chemotherapy of Cancer Dissemination and Metastasis*, (Eds. Carrattini and Franchi) p. 71. New York: Raven Press.

FRANKS, L.M., CARBONELL, A.W., HEMMINGS, V.J. & RIDDLE, P.N. (1976). Metastasizing tumours from serum-supplemented and serum-free lines from a C57BL mouse lung tumour. *Cancer Res.*, **36**, 1049.

KINSEY, D.L. (1960). An experimental study of preferential metastasis. *Cancer*, **13**, 674.

KRAMER, R.H. & NICOLSON, G.L. (1979). Interactions of tumour cells with vascular endothelial cell monolayers: A model for metastatic invasion. *Proc. Nat Acad. Sci.*, **76**, 5704.

LAYTON, M.G. & FRANKS, L.M. (1984). Heterogeneity in a spontaneous mouse lung carcinoma: Selection and characterisation of stable metastatic variants. *Br. J. Cancer*, **49**, 00.

MURRAY, J.C., GARBISA, S. & LIOTTA, L. (1980). The role of tumour cell-basement membrane interactions in the metastatic process. In *Metastasis – Clinical and Experimental Aspects*. (Eds. Hellman *et al.*) *Dev. Oncol.* **4**, 169.

NICOLSON, G.L. (1982). Cancer metastasis – organ colonization and the cell surface properties of malignant cells. *Biochem. Biophys. Acta*, **695**, 113.

PAGET, S. (1899). The distribution of secondary growth in cancer of the breast. *Lancet*, **i**, 571.

ROWLATT, C., FRANKS, L.M., SHERIFF, M.U. & CHESTERMAN, F.C. (1969). Naturally occurring tumours and other lesions of the digestive tract in untreated C57BL mice. *J. Nat Cancer Inst.*, **43**, 1353.

WEXLER, H. (1966). Accurate identification of experimental pulmonary metastases. *J. Nat Cancer Inst.*, **36**, 641.

WILLIS, R.A. (1967). *Pathology of Tumours*. 4th edition. Butterworths.